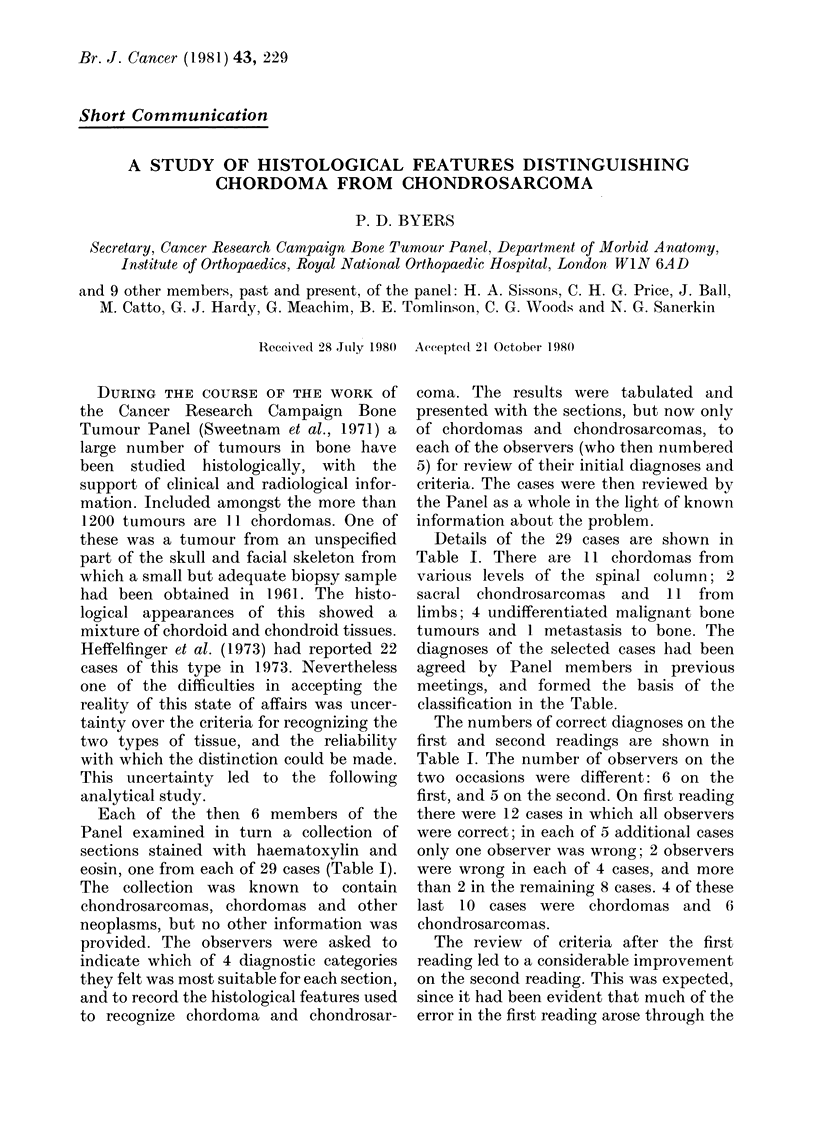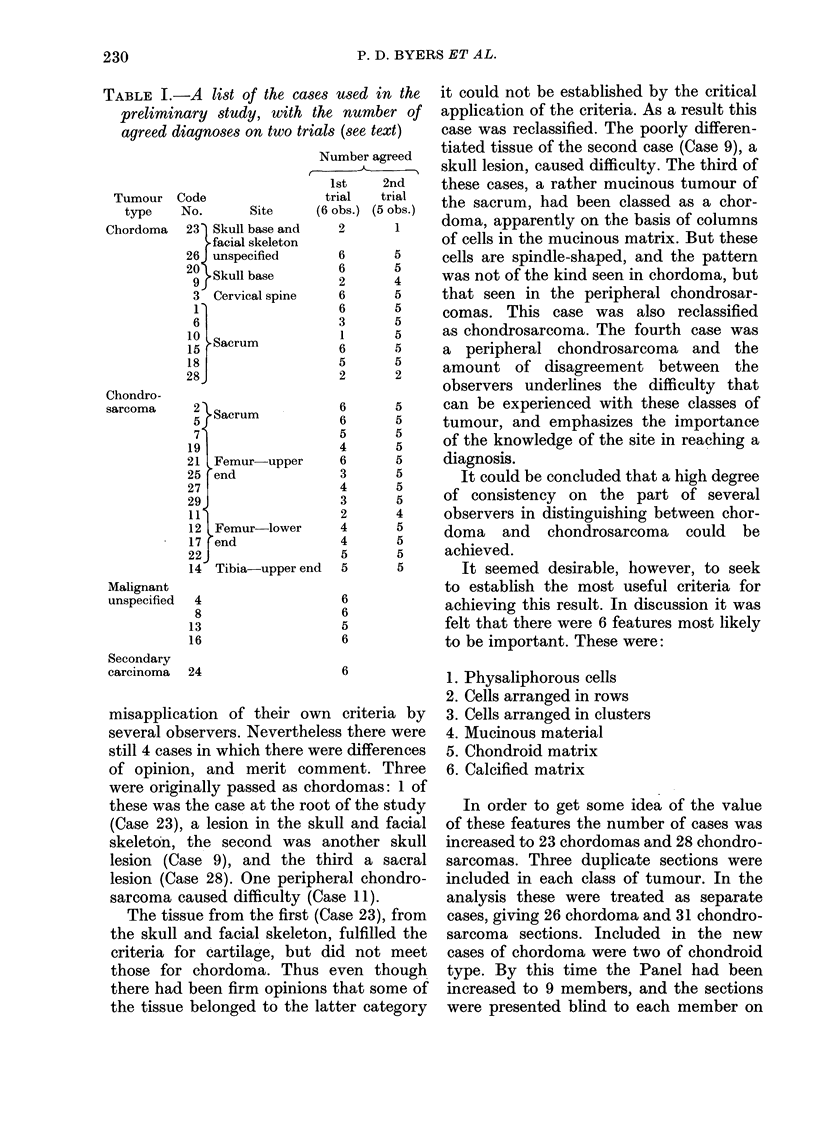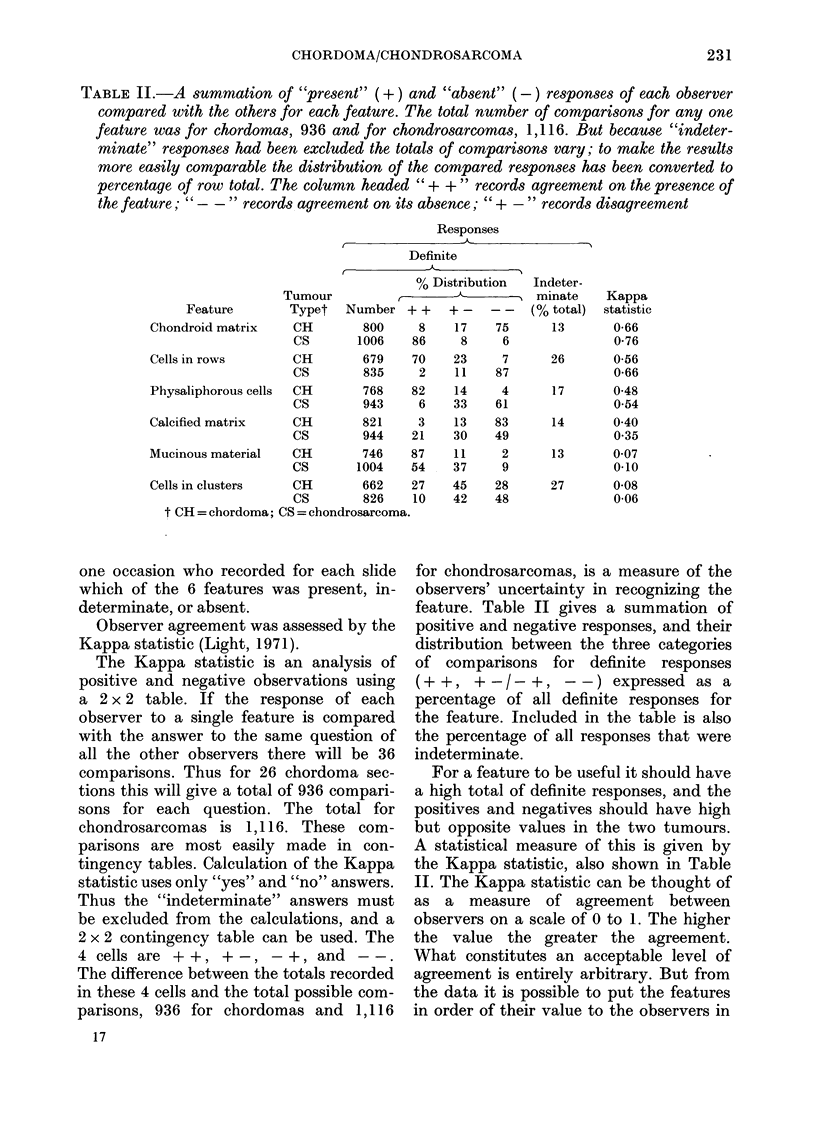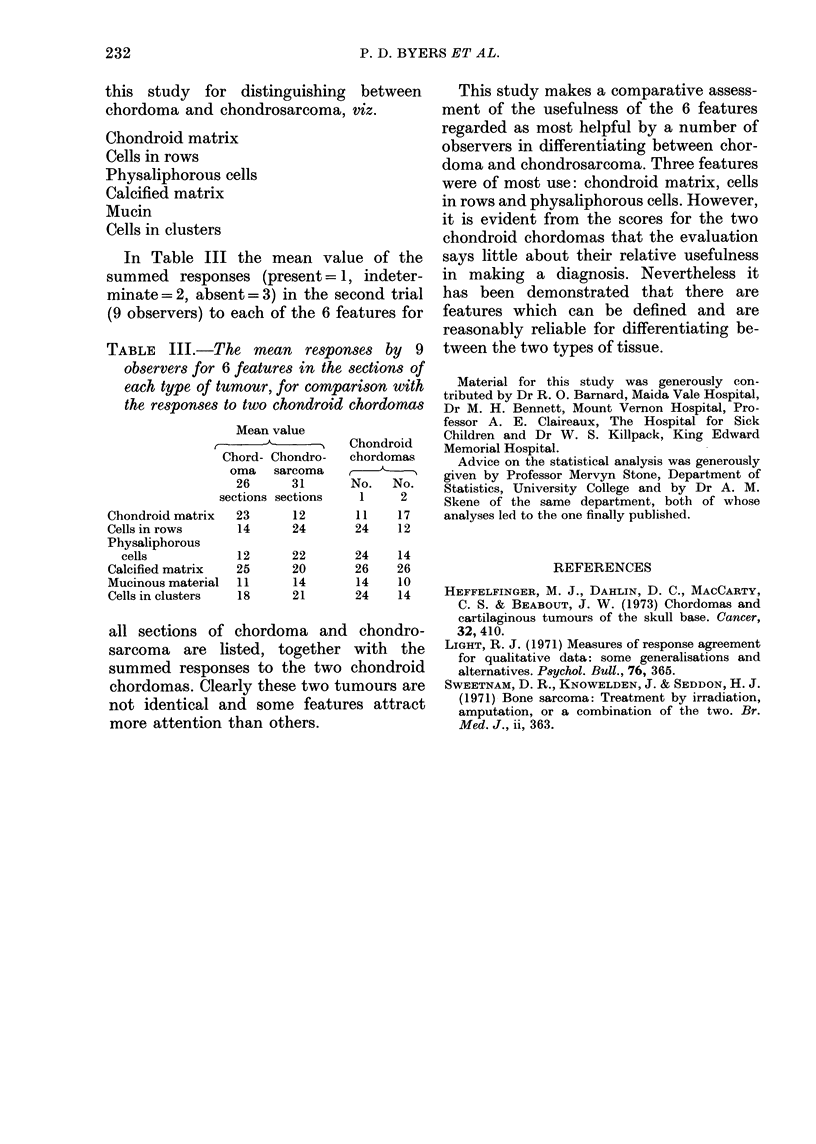# A study of histological features distinguishing chordoma from chondrosarcoma.

**DOI:** 10.1038/bjc.1981.33

**Published:** 1981-02

**Authors:** P. D. Byers


*Br. J. Cancer* (1981) **43**, 229

***Short Communication***

### A STUDY OF HISTOLOGICAL FEATURES DISTINGUISHING CHORDOMA FROM CHONDROSARCOMA

P. D. BYERS

*Secretary, Cancer Research Campaign Bone Tumour Panel, Department of Morbid Anatomy, Institute of Orthopaedics, Royal National Orthopaedic Hospital, London W1N 6AD*

and 9 other members, past and present, of the panel: H. A. Sissons, C. H. G. Price, J. Ball, M. Catto, G. J. Hardy, G. Meachim, B. E. Tomlinson, C. G. Woods and N. G. Sanerkin

Received 28 July 1980 Accepted 21 October 1980

DURING THE COURSE OF THE WORK of the Cancer Research Campaign Bone Tumour Panel (Sweetnam *et al.*, 1971) a large number of tumours in bone have been studied histologically, with the support of clinical and radiological information. Included amongst the more than 1200 tumours are 11 chordomas. One of these was a tumour from an unspecified part of the skull and facial skeleton from which a small but adequate biopsy sample had been obtained in 1961. The histological appearances of this showed a mixture of chordoid and chondroid tissues. Heffelfinger *et al.* (1973) had reported 22 cases of this type in 1973. Nevertheless one of the difficulties in accepting the reality of this state of affairs was uncertainty over the criteria for recognizing the two types of tissue, and the reliability with which the distinction could be made. This uncertainty led to the following analytical study.

Each of the then 6 members of the Panel examined in turn a collection of sections stained with haematoxylin and eosin, one from each of 29 cases (Table I). The collection was known to contain chondrosarcomas, chordomas and other neoplasms, but no other information was provided. The observers were asked to indicate which of 4 diagnostic categories they felt was most suitable for each section, and to record the histological features used to recognize chordoma and chondrosarcoma. The results were tabulated and presented with the sections, but now only of chordomas and chondrosarcomas, to each of the observers (who then numbered 5) for review of their initial diagnoses and criteria. The cases were then reviewed by the Panel as a whole in the light of known information about the problem.

Details of the 29 cases are shown in Table I. There are 11 chordomas from various levels of the spinal column; 2 sacral chondrosarcomas and 11 from limbs; 4 undifferentiated malignant bone tumours and 1 metastasis to bone. The diagnoses of the selected cases had been agreed by Panel members in previous meetings, and formed the basis of the classification in the Table.

The numbers of correct diagnoses on the first and second readings are shown in Table I. The number of observers on the two occasions were different: 6 on the first, and 5 on the second. On first reading there were 12 cases in which all observers were correct; in each of 5 additional cases only one observer was wrong; 2 observers were wrong in each of 4 cases, and more than 2 in the remaining 8 cases. 4 of these last 10 cases were chordomas and 6 chondrosarcomas.

The review of criteria after the first reading led to a considerable improvement on the second reading. This was expected, since it had been evident that much of the error in the first reading arose through the

TABLE I.—*A list of the cases used in the preliminary study, with the number of agreed diagnoses on two trials (see text)*

| Tumour type | Code No. | Site | Number agreed: 1st trial (6 obs.) | Number agreed: 2nd trial (5 obs.) |
|---|---|---|---|---|
| Chordoma | 23 | Skull base and facial skeleton unspecified | 2 | 1 |
| | 26 | | 6 | 5 |
| | 20 | Skull base | 6 | 5 |
| | 9 | | 2 | 4 |
| | 3 | Cervical spine | 6 | 5 |
| | 1 | Sacrum | 6 | 5 |
| | 6 | | 3 | 5 |
| | 10 | | 1 | 5 |
| | 15 | | 6 | 5 |
| | 18 | | 5 | 5 |
| | 28 | | 2 | 2 |
| Chondro-sarcoma | 2 | Sacrum | 6 | 5 |
| | 5 | | 6 | 5 |
| | 7 | Femur—upper end | 5 | 5 |
| | 19 | | 4 | 5 |
| | 21 | | 6 | 5 |
| | 25 | | 3 | 5 |
| | 27 | | 4 | 5 |
| | 29 | | 3 | 5 |
| | 11 | Femur—lower end | 2 | 4 |
| | 12 | | 4 | 5 |
| | 17 | | 4 | 5 |
| | 22 | | 5 | 5 |
| | 14 | Tibia—upper end | 5 | 5 |
| Malignant unspecified | 4 | | 6 | |
| | 8 | | 6 | |
| | 13 | | 5 | |
| | 16 | | 6 | |
| Secondary carcinoma | 24 | | 6 | |

misapplication of their own criteria by several observers. Nevertheless there were still 4 cases in which there were differences of opinion, and merit comment. Three were originally passed as chordomas: 1 of these was the case at the root of the study (Case 23), a lesion in the skull and facial skeleton, the second was another skull lesion (Case 9), and the third a sacral lesion (Case 28). One peripheral chondrosarcoma caused difficulty (Case 11).

The tissue from the first (Case 23), from the skull and facial skeleton, fulfilled the criteria for cartilage, but did not meet those for chordoma. Thus even though there had been firm opinions that some of the tissue belonged to the latter category it could not be established by the critical application of the criteria. As a result this case was reclassified. The poorly differentiated tissue of the second case (Case 9), a skull lesion, caused difficulty. The third of these cases, a rather mucinous tumour of the sacrum, had been classed as a chordoma, apparently on the basis of columns of cells in the mucinous matrix. But these cells are spindle-shaped, and the pattern was not of the kind seen in chordoma, but that seen in the peripheral chondrosarcomas. This case was also reclassified as chondrosarcoma. The fourth case was a peripheral chondrosarcoma and the amount of disagreement between the observers underlines the difficulty that can be experienced with these classes of tumour, and emphasizes the importance of the knowledge of the site in reaching a diagnosis.

It could be concluded that a high degree of consistency on the part of several observers in distinguishing between chordoma and chondrosarcoma could be achieved.

It seemed desirable, however, to seek to establish the most useful criteria for achieving this result. In discussion it was felt that there were 6 features most likely to be important. These were:

1. Physaliphorous cells
2. Cells arranged in rows
3. Cells arranged in clusters
4. Mucinous material
5. Chondroid matrix
6. Calcified matrix

In order to get some idea of the value of these features the number of cases was increased to 23 chordomas and 28 chondrosarcomas. Three duplicate sections were included in each class of tumour. In the analysis these were treated as separate cases, giving 26 chordoma and 31 chondrosarcoma sections. Included in the new cases of chordoma were two of chondroid type. By this time the Panel had been increased to 9 members, and the sections were presented blind to each member on

TABLE II.—*A summation of "present" (+) and "absent" (−) responses of each observer compared with the others for each feature. The total number of comparisons for any one feature was for chordomas,* 936 *and for chondrosarcomas,* 1,116. *But because "indeterminate" responses had been excluded the totals of comparisons vary; to make the results more easily comparable the distribution of the compared responses has been converted to percentage of row total. The column headed "+ +" records agreement on the presence of the feature; "− −" records agreement on its absence; "+ −" records disagreement*

| | | Responses | | | | | |
|---|---|---|---|---|---|---|---|
| | | Definite | | | | | |
| | | | % Distribution | | | | |
| Feature | Tumour Type† | Number | + + | + − | − − | Indeterminate (% total) | Kappa statistic |
| Chondroid matrix | CH | 800 | 8 | 17 | 75 | 13 | 0·66 |
| | CS | 1006 | 86 | 8 | 6 | | 0·76 |
| Cells in rows | CH | 679 | 70 | 23 | 7 | 26 | 0·56 |
| | CS | 835 | 2 | 11 | 87 | | 0·66 |
| Physaliphorous cells | CH | 768 | 82 | 14 | 4 | 17 | 0·48 |
| | CS | 943 | 6 | 33 | 61 | | 0·54 |
| Calcified matrix | CH | 821 | 3 | 13 | 83 | 14 | 0·40 |
| | CS | 944 | 21 | 30 | 49 | | 0·35 |
| Mucinous material | CH | 746 | 87 | 11 | 2 | 13 | 0·07 |
| | CS | 1004 | 54 | 37 | 9 | | 0·10 |
| Cells in clusters | CH | 662 | 27 | 45 | 28 | 27 | 0·08 |
| | CS | 826 | 10 | 42 | 48 | | 0·06 |

† CH = chordoma; CS = chondrosarcoma.

one occasion who recorded for each slide which of the 6 features was present, indeterminate, or absent.

Observer agreement was assessed by the Kappa statistic (Light, 1971).

The Kappa statistic is an analysis of positive and negative observations using a 2 × 2 table. If the response of each observer to a single feature is compared with the answer to the same question of all the other observers there will be 36 comparisons. Thus for 26 chordoma sections this will give a total of 936 comparisons for each question. The total for chondrosarcomas is 1,116. These comparisons are most easily made in contingency tables. Calculation of the Kappa statistic uses only "yes" and "no" answers. Thus the "indeterminate" answers must be excluded from the calculations, and a 2 × 2 contingency table can be used. The 4 cells are + +, + −, − +, and − −. The difference between the totals recorded in these 4 cells and the total possible comparisons, 936 for chordomas and 1,116 for chondrosarcomas, is a measure of the observers' uncertainty in recognizing the feature. Table II gives a summation of positive and negative responses, and their distribution between the three categories of comparisons for definite responses (+ +, + −/− +, − −) expressed as a percentage of all definite responses for the feature. Included in the table is also the percentage of all responses that were indeterminate.

For a feature to be useful it should have a high total of definite responses, and the positives and negatives should have high but opposite values in the two tumours. A statistical measure of this is given by the Kappa statistic, also shown in Table II. The Kappa statistic can be thought of as a measure of agreement between observers on a scale of 0 to 1. The higher the value the greater the agreement. What constitutes an acceptable level of agreement is entirely arbitrary. But from the data it is possible to put the features in order of their value to the observers in this study for distinguishing between chordoma and chondrosarcoma, *viz.*

Chondroid matrix
Cells in rows
Physaliphorous cells
Calcified matrix
Mucin
Cells in clusters

In Table III the mean value of the summed responses (present = 1, indeterminate = 2, absent = 3) in the second trial (9 observers) to each of the 6 features for all sections of chordoma and chondrosarcoma are listed, together with the summed responses to the two chondroid chordomas. Clearly these two tumours are not identical and some features attract more attention than others.

TABLE III.—*The mean responses by 9 observers for 6 features in the sections of each type of tumour, for comparison with the responses to two chondroid chordomas*

| | Mean value | | Chondroid chordomas | |
|---|---|---|---|---|
| | Chordoma 26 sections | Chondrosarcoma 31 sections | No. 1 | No. 2 |
| Chondroid matrix | 23 | 12 | 11 | 17 |
| Cells in rows | 14 | 24 | 24 | 12 |
| Physaliphorous cells | 12 | 22 | 24 | 14 |
| Calcified matrix | 25 | 20 | 26 | 26 |
| Mucinous material | 11 | 14 | 14 | 10 |
| Cells in clusters | 18 | 21 | 24 | 14 |

This study makes a comparative assessment of the usefulness of the 6 features regarded as most helpful by a number of observers in differentiating between chordoma and chondrosarcoma. Three features were of most use: chondroid matrix, cells in rows and physaliphorous cells. However, it is evident from the scores for the two chondroid chordomas that the evaluation says little about their relative usefulness in making a diagnosis. Nevertheless it has been demonstrated that there are features which can be defined and are reasonably reliable for differentiating between the two types of tissue.

Material for this study was generously contributed by Dr R. O. Barnard, Maida Vale Hospital, Dr M. H. Bennett, Mount Vernon Hospital, Professor A. E. Claireaux, The Hospital for Sick Children and Dr W. S. Killpack, King Edward Memorial Hospital.

Advice on the statistical analysis was generously given by Professor Mervyn Stone, Department of Statistics, University College and by Dr A. M. Skene of the same department, both of whose analyses led to the one finally published.

### REFERENCES

HEFFELFINGER, M. J., DAHLIN, D. C., MACCARTY, C. S. & BEABOUT, J. W. (1973) Chordomas and cartilaginous tumours of the skull base. *Cancer*, **32**, 410.

LIGHT, R. J. (1971) Measures of response agreement for qualitative data: some generalisations and alternatives. *Psychol. Bull.*, **76**, 365.

SWEETNAM, D. R., KNOWELDEN, J. & SEDDON, H. J. (1971) Bone sarcoma: Treatment by irradiation, amputation, or a combination of the two. *Br. Med. J.*, ii, 363.